# Single Nucleotide Polymorphisms in the *FADS* Gene Cluster but not the *ELOVL2* Gene are Associated with Serum Polyunsaturated Fatty Acid Composition and Development of Allergy (in a Swedish Birth Cohort)

**DOI:** 10.3390/nu7125521

**Published:** 2015-12-03

**Authors:** Malin Barman, Staffan Nilsson, Åsa Torinsson Naluai, Anna Sandin, Agnes E. Wold, Ann-Sofie Sandberg

**Affiliations:** 1 Department of Biology and Biological Engineering, Food and Nutrition Science, Chalmers University of Technology, 412 96 Göteborg, Sweden; ann-sofie.sandberg@chalmers.se; 2 Department of Mathematical Sciences, Chalmers University of Technology, 412 96 Göteborg, Sweden; Staffan.nilsson@chalmers.se; 3 Department of Microbiology and Immunology, Institute of Biomedicine, University of Gothenburg, 405 30 Göteborg, Sweden; asa@genomics.sahlgrenska.gu.se; 4 Department of Clinical Sciences, Paediatrics, Umeå University, 901 87 Umeå, Sweden; kxanasan@nll.se; 5 Department of Infectious Diseases, Institute of Biomedicine, University of Gothenburg, 405 30 Göteborg, Sweden; agnes.wold@microbio.se

**Keywords:** long chain polyunsaturated fatty acids, arachidonic acid, phospholipids, umbilical cord serum, single nucleotide polymorphism, fatty acid desaturase, *FADS*, elongase, *ELOVL2*, nutrigenetics, allergy, atopic eczema, respiratory allergy, BAS birth cohort

## Abstract

Exposure to polyunsaturated fatty acids (PUFA) influences immune function and may affect the risk of allergy development. Long chain PUFAs are produced from dietary precursors catalyzed by desaturases and elongases encoded by *FADS* and *ELOVL* genes. In 211 subjects, we investigated whether polymorphisms in the *FADS* gene cluster and the *ELOVL2* gene were associated with allergy or PUFA composition in serum phospholipids in a Swedish birth-cohort sampled at birth and at 13 years of age; allergy was diagnosed at 13 years of age. Minor allele carriers of rs102275 and rs174448 (*FADS* gene cluster) had decreased proportions of 20:4 *n*-6 in cord and adolescent serum and increased proportions of 20:3 *n*-6 in cord serum as well as a nominally reduced risk of developing atopic eczema, but not respiratory allergy, at 13 years of age. Minor allele carriers of rs17606561 in the *ELOVL2* gene had nominally decreased proportions of 20:4 *n*-6 in cord serum but *ELOVL* polymorphisms (rs2236212 and rs17606561) were not associated with allergy development. Thus, reduced capacity to desaturase *n*-6 PUFAs due to *FADS* polymorphisms was nominally associated with reduced risk for eczema development, which could indicate a pathogenic role for long-chain PUFAs in allergy development.

## 1. Introduction

**Figure 1:**
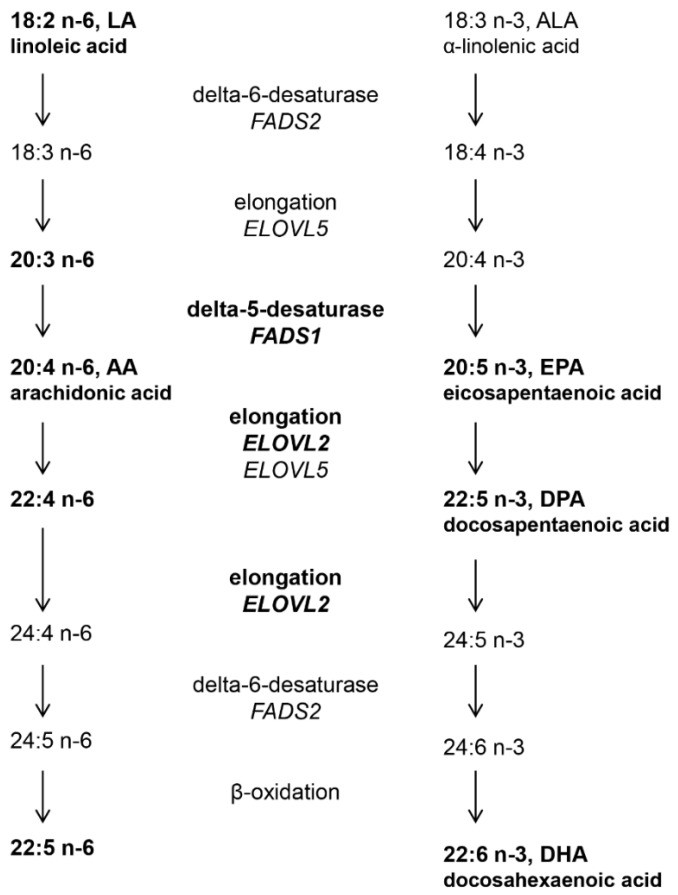
The metabolic pathways of polyunsaturated fatty acids in mammals [26]. Fatty acids and genes in bold were examined in this study.

Polyunsaturated fatty acids (PUFAs) are essential for cell and tissue development and a sufficient supply of PUFA is important from fetal life onwards. The fetus is mainly supplied with PUFAs by transfer from the maternal circulation via the placenta [1]. After birth, PUFAs are available via the diet, including breast milk or formula in infants and from fatty foods later in life. Important long chain PUFAs, such as arachidonic acid and docosahexaenoic acid (DHA) may also be produced in the body from their essential precursor fatty acids linoleic acid (18:2 *n*-6) and α-linolenic acid (18:3 *n*-3) that are abundant in the diet [2]. Substrate fatty acids are elongated by sequential addition of two-carbon atom units and desaturated by introduction of a double bond in the molecule. The elongation step is catalyzed by elongases encoded by the *ELOVL* (elongation of very long chain fatty acids) gene family on chromosome 6 [3], while desaturation is catalyzed by desaturases such as Δ-5 and Δ-6 desaturases, encoded by the *FADS* (fatty acid desaturase) gene cluster on chromosome 11 [4,5,6] (Figure 1). The production of longer *n*-6 and longer *n*-3 PUFAs involves the same enzymes (elongases and desaturases), hence, there is a competition for the enzymes between the two pathways (Figure 1). Desaturation is the rate limiting step in this pathway and several studies have revealed that single nucleotide polymorphism (SNPs) in the *FADS* gene cluster affect the proportions of PUFA and long chain PUFA in human tissue [7,8,9,10,11,12,13,14,15,16,17,18,19,20,21,22,23]. Polymorphism in the *FADS2* gene that negatively affect the activity of the Δ-6 desaturase, have been associated with increased proportions of linoleic acid (precursor for the *n*-6 series) and α-linolenic acid (precursor for the *n*-3 series), while the products arachidonic acid (*n*-6) and eicosapentaenoic acid (EPA, *n*-3) are reduced. Recent genome-wide association studies have also suggested that polymorphism in the *ELOVL2* gene are associated with increased proportions of the substrate and decreased proportions of the products [23,24,25].

PUFAs are important modulators of immune function. Long chain PUFAs are powerful inhibitors of mitogen-induced activation [27,28,29] and secretion of interferon-γ by T cells [30,31]. Furthermore, the long chain *n*-6 PUFA arachidonic acid is the precursor of prostaglandins [32] that promote maturation of dendritic cells into a phenotype that favors Th2 lineage commitment from naïve T cells with which the dendritic cell interacts. Th2 cells are central in atopic (IgE-mediated) allergy. We have recently shown that the risk of developing allergy is positively related to a high proportion of both *n*-6 and *n*-3 long chain PUFAs in cord blood [33]. Since variation in the *FADS* genes affects serum proportions of long chain PUFAs [24,25,34] we speculated that genetic variation in the *FADS* genes may also affect the risk of allergy development. Indeed, a German study found that carriers of the minor alleles of several SNPs in *FADS* and their respective haplotypes had a lower prevalence of allergic rhinitis and atopic eczema [13]. The effect of *ELOVL* polymorphisms on risk of allergy development has not previously been studied.

The aim of the present study was to evaluate if genetic variations in the *FADS* gene cluster or in the *ELOVL2* gene were associated with proportions of long chain PUFAs in cord serum phospholipids or in adolescents’ serum phospholipids, and to examine any potential association between genetic variation and allergic disease at 13 years of age.

## 2. Experimental Section

### 2.1. Subjects

The birth-cohort consisted of all children (*N* = 1228) born during one year between February 1996 and January 1997 at Östersund Hospital in the County of Jämtland in Northern Sweden. The cohort was designed to assess the development of allergy and the children underwent skin prick tests at one and four years of age to detect sensitization to common food and airborne allergens. Also, their parents responded to questionnaires regarding the children’s allergic symptoms at one, four, and seven years of age. Socioeconomic, housing, and lifestyle factors were recorded by questionnaires. As previously described [35,36], adolescents still participating in the study at 13 years of age (*n* = 841) were invited to fill out a questionnaire regarding allergy symptoms, and other environmental factors known to influence allergy, take part in skin prick tests, and donate a blood sample for PUFA analysis. Seven hundred and eighty eight of the 841 adolescents took part in both skin prick test and answered the questionnaire at the follow up at 13 years of age.

### 2.2. Allergy Diagnosis

Based on the questionnaires and sensitization tests at 13 years of age, the subjects were divided into three diagnostic groups: (1) atopic eczema with no other allergic manifestations, in all, 79 adolescents out of the 788 fulfilled these criteria; (2) respiratory allergy with no other allergic manifestations, all of whom were also sensitized to airborne allergens, in all, 130 adolescents in the cohort fulfilled these criteria and (3) no allergy and no sensitization at any of the follow-ups (1, 4, 7 and 13 years of age), in all, 331 adolescents in the cohort fulfilled these criteria. Atopic eczema was defined as a pruritic, chronic, or chronically relapsing non-infectious dermatitis with typical features and distribution, fulfilling three of the main criteria suggested by Hanifin and Rajka [37]. Respiratory allergy was defined as a positive skin prick test to an inhalant allergen, in combination with two or more of the following criteria: wheeze in the past year, doctor’s diagnosed asthma, asthma inhalation treatment, or a positive answer to the question: “Have you had any signs of pollen allergy or allergy to furry pets during the last 12 months?”.

### 2.3. Collection of Serum

Cord blood samples, a mixture of arterial and venous blood, were obtained at delivery from 819 of the children born vaginally. Serum was separated, aliquoted, and stored frozen until analyzed. At 13 years of age, a subgroup of 300 adolescents was invited to take part in a new blood sampling. Two hundred and seventy two adolescents gave blood on three different occasions: November 2009 (*n* = 88), April 2010 (*n* = 52) and January 2011 (*n* = 132). Venous blood, 10 mL, was drawn at any time of the day, fasting was not required. The blood was allowed to clot. Serum was separated by centrifugation, aliquoted and frozen within 3 h. It was stored at −80 °C until analyzed.

**Table 1:** Characteristics of the birth cohort and the study population.

			Selected Subjects ^2^		
	All Subjects ^1^	Selected Subjects ^2^	No Allergy ^3^	Atopic Eczema ^4^	Respiratory Allergy ^5^
	*n* = 841	*n* = 211	*n* = 88	*n* = 41	*n* = 82
Antenatal characteristics					
Heridity					
Maternal, %	43	45	36	51	52
Paternal, %	36	40	31	37	51
Maternal age at delivery ^6^, years old	29 (15–45)	29 (19–43)	29 (19–42)	29 (21–43)	29 (21–43)
Siblings ^6^, %	60	60	59	62	61
Birth characteristics					
Gestational age at delivery ^6^, weeks	40 (30–43)	40 (34–43)	39.6 (35–43)	39.8 (34–43)	40.0 (35–42)
Birth weight ^6^, g	3573 (1386–5400)	3632 (2485–5400)	3606 (2485–5320)	3580 (2575–4300)	3685 (2588–5400)
Infant characteristic					
Male gender, %	50	47	43	29	60
Exclusive breastfeeding at 4 months, %	72	72	74	77	68
Adolescent characteristics					
BMI	19 (14–33)	19 (14–29)	20 (15–27)	19 (15–25)	19 (14–29)
Sensitized	32	41	0	10	100

Data are presented as % or mean (range). ^1^ All subjects that were invited to take part in the follow up at 13 years of age; ^2^ Subjects that were selected for blood sampling at 13 years of age and approved participation in genetic analyses; ^3^ No allergy and no sensitization at any of the follow-ups (1, 4, 7, and 13 years of age); ^4^ Atopic eczema was defined as a pruritic, chronic, or chronically relapsing non-infectious dermatitis with typical features and distribution, fulfilling three of the main criteria suggested by Hanifin and Rajka 6; ^5^ Respiratory allergy was defined as a positive skin prick test to an inhalant allergen, in combination with two or more of the following criteria: wheeze in the past year, doctor’s diagnosed asthma, asthma inhalation treatment, or a positive answer to the question: “Have you had any signs of pollen allergy or allergy to furry pets during the last 12 months?”; ^6^ Data obtained from the Swedish Medical Birth Register kept by The Swedish National Board of Health and Welfare (Stockholm, Sweden).

### 2.4. Selection of Subjects

Out of the 272 subjects that agreed to give a blood sample at 13 years of age, 261 subjects gave blood for genetic analysis. From these, 211 subjects were selected for analysis of genetic polymorphisms, based on availability of cord serum. Forty one of these subjects had atopic eczema, 82 had respiratory allergy, and 88 were non-allergic and non-sensitized.

### 2.5. Analysis of Fatty Acids in Serum Phospholipids

Briefly, 200 µL of serum was thawed in room temperature, vortexed and mixed with chloroform:methanol (1:2) and 0.5% NaCl-solution [38]. Phospholipids were obtained by separation on aminopropyl solid phase extraction columns (Isolute NH2, 6 mL, 500 mg, IST, Mid Glamorgon, Cardiff, UK) [39] and converted to fatty acid methyl esters using acetyl chloride (10%) dissolved in methanol [40] during overnight incubation in room temperature. The fatty acid methyl esters were then extracted with 2 mL of petroleum ether. After evaporation under N_2_ in a 40 °C water bath the samples were re-dissolved in 200 µL isooctane and fatty acid methyl esters were separated by gas chromatography (Hewlett Packard 5890, Agilent Technology., Waldbronn, Germany). Detection was done by flame ionization and the Borwin software ((JMBS Developpements, Le Fontanil, France) was used for evaluation. The fatty acids were separated by gas chromatography on two different columns. The samples were first separated on an HP Ultra 1 (50 m × 0.32 mm × 0.52 µm *df* silicon column (J & W Scientific, Folsom, CA, USA) suitable for separation of the 20–22 carbon atom-long fatty acids. Where sufficient materials remained in the vial after the first GC-run, we also performed separation of 16–18 carbon atom-long fatty acids on a DB-WAX (30 m × 0.25 mm × 0.25 µm *df*) column (J & W Scientific, Folsom, CA, USA). Fatty acids that were found above the limit of quantification and could clearly be separated were selected for statistical analyses. The proportion of each fatty acid was expressed as area percentage of the total fatty acids, *i.e.*, all fatty acids 16–22 carbon atoms long.

### 2.6. Genetic Analyses

**Figure 2:**
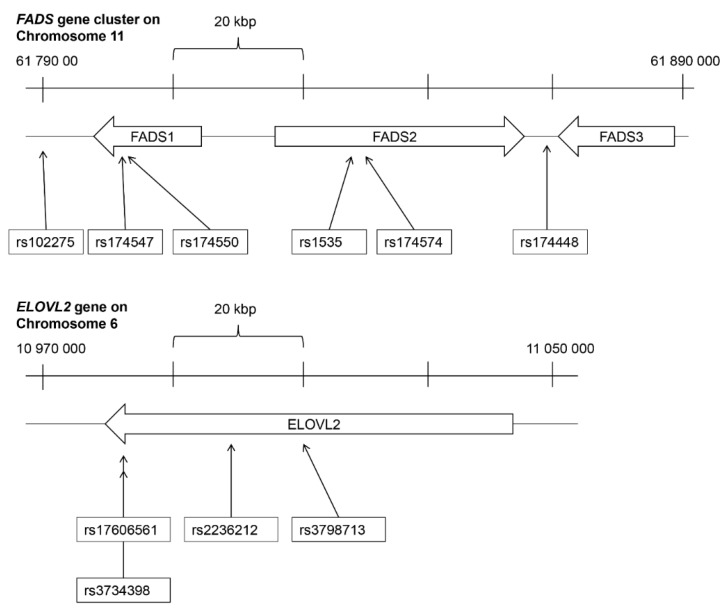
Overview of the *FADS* gene cluster (in top) and the *ELOVL2* gene (below) and the SNPs analyzed in this study. Position was derived from UCSC Genome Browser on Human December 2013 (GRCh38/hg38) Assembly.

Genomic DNA was extracted from venous blood collected in EDTA tubes from the subjects at 13 years of age (*n* = 211). The standard protocol at Kbioscienses (LGC genomics, Hoddesdon, UK) was used. SNP genotyping in the present study was guided by previous genome-wide association studies showing significant association between fatty acids in serum phospholipids and SNPs in the *FADS* gene cluster or the *ELOVL* gene family [24,25]. A total of six SNPs had been identified in the *FADS* gene cluster (rs102275, rs174547, rs174550, rs1535, rs174574, rs14448) and a total of four SNPs had been identified in the *ELOVL* gene family (rs17606561, rs3798713, rs3734398, rs2236212, all situated in the *ELOVL2* gene) [24,25]. For the six identified SNPs in the *FADS* gene cluster rs102275 was in complete linkage disequilibrium (LD, *r*^2^ = 1) with rs1535, rs174574, rs174547, and rs174550, but not with rs174448 and hence only rs102275 and rs174448 were genotyped. For the four SNPs in the *ELOVL2* gene rs2236212 was in complete LD with rs3798713 (*r*^2^ = 1) and in almost complete LD with rs3734398 (*r*^2^ = 0.966), therefore only rs2236212 and rs17606561 were genotyped from these four identified SNPs. This led to two SNPs in the *FADS* gene cluster (rs102275 and rs174448) and two SNPs in the *ELOVL2* gene (rs2236212 and rs17606561) being selected for genotyping (Figure 2). Characteristics of the analyzed SNPs are shown in Table 2. 

**Table 2:** Characteristics of single nucleotide polymorphisms in the *ELOVL2* gene and the *FADS* gene cluster.

SNP	Function	Chromosome	Position ^1^ (bp)	Major > Minor	Minor Allele Frequency ^2^
*ELOVL2* gene					
rs17606561	3 prime UTR variant	6	10 982 126	G > A	0.182
rs2236212	Intron variant	6	10 994 782	G > C	0.487
*FADS* gene cluster					
rs102275	Intergenic	11	61 790 331	T > C	0.493
rs174448	Intergenic	11	61 872 101	A > G	0.404

^1^ Position in base pairs was derived from UCSC Genome Browser on Human December 2013 (GRCh38/hg38) Assembly; ^2^ Minor allele frequency is taken from the current default global population is 1000Genome phase 1 genotype data from 1094 worldwide individuals, released in the May 2011 dataset.

### 2.7. Statistics

Hardy-Weinberg equilibrium was investigated with a chi-square goodness of fit. SNPs were analyzed coded according to minor allele count (0, 1, 2) and analyzed as a numeric variable. The association between SNPs and fatty acids was analyzed using linear regression and the association between SNPs and allergy was analyzed with logistic regression both univariable and in a multivariable setting with a risk associated fatty acid as the other predictor. Haplotypes were constructed using the EM-algorithm and haplotype counts used as predictors. Correction for multiple testing was carried out by permutation [41] to take into account the correlations between outcomes as well as SNPs. For each gene separately and for three separate outcome groups—15 phospholipid variables in cord serum and in adolescent serum and also for the two diagnosis atopic eczema and respiratory allergy—100 000 permutations were carried out to get an empirical null distribution of the minP (minimum *p*-value) statistic from which the corrected p-values could be estimated Two-tailed *p* ≤ 0.05 was considered significant [41]. The statistical analyses were performed using R 2.13.1 (R Foundation for Statistical Computing, Vienna, Austria) [42].

### 2.8. Ethic Statement

The study was conducted according to the Helsinki II Declaration [43] and was approved by the local ethical committee in Umeå, Sweden (Dnr 95–49, 09–017 M and 09–110 M). Pregnant women were recruited in their 18th gestational week. Participation was voluntary and those mothers who accepted to participate in the study provided written consent forms concerning collection of cord blood. Thirteen years later, on behalf of the minors, parents of the adolescents provided a new written consent form and the adolescents themselves approved their participation orally for skin prick tests and for fatty acid analyses in cord serum as well as in the new serum samples. Participation was voluntary and the adolescents were free to decide not to participate in further tests and questionnaires without any stated reason.

## 3. Results

### 3.1. Fatty Acid Proportions in Serum Phospholipids

Proportions of PUFAs were analyzed in the phospholipid fraction of infant cord and adolescents’ sera. Table 3 shows median proportions of the nine PUFAs that were analyzed in relation to polymorphism in this study.

**Table 3:** Fatty acid proportions (% of total fatty acids) in serum phospholipids at birth and at 13 years of age.

	Serum Phospholipid Proportion Median (Intraquartile Range)	
Fatty Acid	Birth (*n* = 118)	13 Years of Age (*n* = 120)
*n*-6		
18:2, linoleic	7.9 (7.2–8.9)	23 (21–26)
20:2, eicosadienoic	0.38 (0.34–0.44)	0.31 (0.26–0.37)
20:3, dihomo-γ-linolenic	5.6 (5.1–6.1)	3.9 (3.2–4.8)
20:4, arachidonic	12 (11–13)	10 (8.4–12)
22:4, adrenic	0.56 (0.48–0.62)	0.36 (0.30–0.44)
22:5, osbond	0.41 (0.32–0.50)	0.20 (0.14–0.30)
*n*-3		
20:5, eicosapentaenoic (EPA)	0.27 (0.20–0.36)	1.1 (0.82–1.4)
22:5, docosapentaenoic (DPA)	0.39 (0.29–0.51)	1.2 (1.1–1.5)
22:6, docosahexaenoic (DHA)	4.4 (3.5–5.0)	4.1 (3.4–4.8)

Proportions of fatty acids were expressed as area percentage of total phospholipid fatty acids, 16–22 carbon long.

### 3.2. Genotyping

The call rate was above 98% in all four SNPs and they all conformed to Hardy-Weinberg equilibrium (*p* > 0.2). The LD between the two *FADS* SNPs was (*r*^2^ = 0.53, *D’* = 0.75) and LD between the two *ELOVL* SNPs was (*r*^2^ = 0.43, *D’* = 1.0). The minor allele frequency in non-allergic and allergic subjects in this study are shown in Table 4.

**Table 4:** Minor allele frequency in non-allergic and allergic subjects in this study.

		Minor Allele Frequency		
SNP	Major > Minor	No Allergy (*n* = 88)	Atopic Eczema (*n* = 41)	Respiratory Allergy (*n* = 82)
*ELOVL2 gene*				
rs17606561	G > A	0.27	0.22	0.23
rs2236212	G > C	0.38	0.40	0.47
*FADS gene cluster*				
rs102275	T > C	0.39	0.25	0.39
rs174448	A > G	0.36	0.21	0.40

Minor allele frequency in the 211 subjects that were genotyped in this study.

### 3.3. Association between Polymorphism in the FADS Gene Cluster and Fatty Acids in Cord Serum

Table 5 shows the association between PUFA proportions measured at birth and polymorphism in the *FADS* gene cluster and *ELOVL2* gene. The associations that were still significant after correction for multiple testing are denoted with stars in Table 5 and are mentioned here. Subjects carrying the minor allele of the two SNPs in the *FADS* gene cluster, rs102275 and rs174448, had elevated proportions of the *n*-6 PUFA pathway substrate dihomo-γ-linolenic acid (20:3 *n*-6) and decreased proportions of the product arachidonic acid (20:4 *n*-6) in cord serum. Accordingly, the ratio of arachidonic acid over dihomo-γ-linolenic acid (20:4 *n*-6/20:3 *n*-6) was significantly associated with both rs102275 and 174448 (Table 5).

**Table 5:** Associations between cord serum proportions of *n*-6 and *n*-3 PUFAs (% of total FA) and fatty acid desaturase (*FADS*) and elongase (*ELOVL*) polymorphisms.

	*FADS*		*FADS*		*ELOVL2*		*ELOVL2*	
	rs102275 T > C		rs174448 A > G		rs2236212 G > C		rs17606561 G > A	
Fatty Acid	*r*	*p*	*r*	*p*	*r*	*p*	*r*	*p*
*n*-6								
18:2, linoleic	0.17	0.14	0.19	0.09	0.03	0.81	0.00	0.97
20:2, eicosadienoic	−0.07	0.44	−0.05	0.56	−0.01	0.89	−0.03	0.75
20:3, dihomo-γ-linolenic	0.50	<0.001 ***	0.35	<0.001 **	−0.09	0.34	−0.04	0.66
20:4, arachidonic	−0.36	<0.001 **	−0.34	<0.001 **	−0.05	0.62	−0.19	0.042
22:4, adrenic	−0.25	0.006	−0.26	0.005	0.09	0.35	−0.10	0.28
22:5, osbond	−0.16	0.09	−0.14	0.14	−0.12	0.20	−0.18	0.06
20:3/18:2 (*FADS2*)	0.24	0.033	0.09	0.42	−0.19	0.09	−0.08	0.48
20:4/20:3 (*FADS1*)	−0.54	<0.001 ***	−0.43	<0.001 ***	0.04	0.69	−0.09	0.32
22:4/20:4 (*ELOVL2*)	0.02	0.87	−0.01	0.92	0.15	0.10	0.04	0.66
22:5/22:4 (*ELOVL2* and *FADS2*)	−0.06	0.54	−0.04	0.63	−0.19	0.039	−0.14	0.13
*n*-3								
20:5, eicosapentaenoic (EPA)	−0.20	0.08	−0.11	0.32	0.02	0.83	−0.00	0.99
22:5, docosapentaenoic (DPA)	−0.06	0.52	−0.04	0.65	0.12	0.20	0.00	0.96
22:6, docosahexaenoic (DHA)	−0.15	0.12	−0.15	0.11	0.02	0.81	−0.14	0.12
22:5/20:5 (*ELOVL2*)	−0.06	0.60	−0.12	0.28	0.06	0.59	0.04	0.71
22:6/22:5 (*ELOVL2* and *FADS2*)	0.01	0.91	0.12	0.19	−0.19	0.038	−0.15	0.10

The associations between SNPs and fatty acids were analyzed using linear regression. SNPs were coded according to minor allele count and analyzed as a numeric variable. Fatty acids were expressed as area percentage of total phospholipid fatty acids, 16–22 carbon long. Abbreviations: *FADS* = fatty acid desaturase, *ELOVL* = elongation of very long chain fatty acids. Correction for multiple inference was carried out by permutation for each gene separately and 15 phospholipid variables in cord serum. The associations that were still significant after corrections are denoted by stars: * Corrected *p* = *p*_c_ < 0.05; ** *p*_c_ < 0.01; *** *p*_c_ < 0.001.

### 3.4. Association between Polymorphism in the FADS Gene Cluster and Fatty Acids in Adolescent Serum

Table 6 shows the association between PUFA proportions measured at 13 years of age and polymorphism in the *FADS* gene cluster and *ELOVL2* gene. The associations that were still significant after correction for multiple testing are denoted with stars in Table 6 and are mentioned here. Similar to the findings in cord blood, subjects carrying the minor allele of rs102275 had significantly lower proportions of arachidonic acid (20:4 *n*-6) in serum phospholipids at 13 years of age and a reduced ratio of arachidonic acid over dihomo-γ-linolenic acid (20:4 *n*-6/20:3 *n*-6) (Table 6).

**Table 6:** Associations between 13 years of age serum proportions of *n*-6 and *n*-3 PUFAs (% of total FA) and fatty acid desaturase (*FADS*) and elongase (*ELOVL*) polymorphisms.

	*FADS*		*FADS*		*ELOVL2*		*ELOVL2*	
	rs102275 T > C		rs174448 A > G		rs2236212 G > C		rs17606561 G > A	
Fatty Acids	*r*	*p*	*r*	*p*	*r*	*p*	*r*	*p*
*n*-6								
18:2, linoleic	0.05	0.76	−0.05	0.76	−0.26	0.10	−0.05	0.76
20:2, eicosadienoic	0.18	0.050	0.02	0.85	0.10	0.26	0.12	0.21
20:3, dihomo-γ-linolenic	0.12	0.21	0.11	0.24	−0.02	0.82	−0.02	0.80
20:4, arachidonic	−0.36	<0.001 **	−0.23	0.013	0.03	0.73	0.04	0.64
22:4, adrenic	−0.14	0.13	−0.06	0.49	0.11	0.26	0.05	0.56
22:5, osbond	−0.04	0.63	0.04	0.70	−0.01	0.90	−0.06	0.49
20:3/18:2 (*FADS2*)	0.02	0.90	0.06	0.69	0.22	0.16	0.06	0.71
20:4/20:3 (*FADS1*)	−0.42	<0.001 ***	−0.30	0.001 *	0.04	0.69	0.03	0.78
22:4/20:4 (*ELOVL2*)	0.06	0.50	0.07	0.46	0.10	0.28	0.01	0.95
22:5/22:4 (*ELOVL2* and *FADS2*)	0.02	0.85	0.07	0.43	−0.15	0.11	−0.23	0.014
*n*-3								
20:5, eicosapentaenoic (EPA)	−0.11	0.22	−0.15	0.09	0.07	0.45	0.05	0.60
22:5, docosapentaenoic (DPA)	−0.12	0.18	−0.02	0.84	0.15	0.11	0.11	0.22
22:6, docosahexaenoic (DHA)	−0.01	0.92	−0.02	0.87	0.00	0.99	−0.02	0.86
22:5/20:5 (*ELOVL2*)	0.08	0.38	0.20	0.032	0.01	0.90	−0.04	0.70
22:6/22:5 (*ELOVL2* and *FADS2*)	0.11	0.22	0.03	0.72	−0.16	0.09	−0.10	0.28

The associations between SNPs and fatty acids were analyzed using linear regression. SNPs were coded according to minor allele count and analyzed as a numeric variable. Fatty acids were expressed as area percentage of total phospholipid fatty acids, 16–22 carbon long. Abbreviations: *FADS* = fatty acid desaturase, *ELOVL* = elongation of very long chain fatty acids. Correction for multiple inference was carried out by permutation for each gene separately and 15 phospholipid fatty acid variables in adolescent serum. The associations that were still significant after correction are denoted by stars: * Corrected *p* = *p*_c_ < 0.05; ** *p*_c_ < 0.01; *** *p*_c_ < 0.001.

### 3.5. Association between Polymorphism in the ELOVL2 Gene and Fatty Acids in Cord and Adolescent Serum

Two *ELOVL2* SNPs were analyzed, rs2236212 and rs17606561. In general, the *ELOVL2* SNPs were less strongly associated with serum fatty acid composition than were *FADS* cluster gene SNPs (Table 5 and Table 6). No correlations were significant after correction for multiple testing, but some were nominally significant. For infant cord serum: rs2236212 was nominally weakly negatively associated with the 22:5 *n*-6/22:4 *n*-6 ratio and to the 22:6 *n*-3/22:5 *n*-3 ratio and rs17606561 was weakly negatively associated to the proportion of arachidonic acid (20:4 *n*-6) (Table 5). In adolescents, rs17606561 was nominally negatively associated with the ratio 22:5 *n*-6/22:4 *n*-6 (Table 6).

### 3.6. Association between Polymorphism in FADS and ELOVL Genes and Risk for Atopic Disease

Carriers of one minor allele of the two *FADS* gene cluster SNPs (rs102275 and rs174448) had reduced capacity to desaturate *n*-6 series PUFAs. These minor allele carriers had approximately half the risk of having atopic eczema at 13 years of age, compared to subjects carrying two major alleles (Table 7). Adding gender to the model did not affect this risk (data not shown). While the associations are nonsignificant after correction for four tests, the direction of the association is consistent with the fatty acids being mediators and thus the use of a one sided test would be justified. No association between the *ELOVL2* gene SNPs and risk for atopic eczema was found. Also, no association was found between any of the four SNPs and the risk of developing respiratory allergy (Table 7). We have previously shown that several cord serum phospholipid fatty acids are associated with development of both atopic eczema and respiratory allergy in these children [33]. If any of the fatty acids that are associated with eczema were added to the logistic models shown in Table 7, the significance of rs102275 and rs174448 disappeared (data not shown), suggesting that the *FADS* polymorphisms reduced atopic eczema development by reducing the capacity to produce long-chain PUFAs.

**Table 7:** Association between *FADS* and *ELOVL* genes and atopic eczema and respiratory allergy.

		Logistic Regression			
		Atopic Eczema		Respiratory Allergy	
Gene	SNP (Major > Minor)	OR	*p* (*p*_c_)	OR	*p* (*p*_c_)
*FADS*	rs102275 (T > C)	0.52 (0.28–0.95)	0.035 (0.104)	1.02 (0.65–1.6)	0.94 (1)
*FADS*	rs174448 (A > G)	0.48 (0.26–0.88)	0.018 (0.055)	1.15 (0.76–1.75)	0.52 (0.9)
*ELOVL*	rs2236212 (G > C)	1.08 (0.64–1.8)	0.78 (1)	1.45 (0.94–2.25)	0.10 (0.3)
*ELOVL*	rs17606561 (G > A)	0.77 (0.41–1.44)	0.41 (0.8)	0.80 (0.48–1.32)	0.37 (0.8)

The association between SNPs and allergy was analyzed with logistic regression. SNPs were coded according to minor allele count and analyzed as a numeric variable. Abbreviations: OR = odds ratio per minor allele. Correction for multiple testing was carried out by permutation for each gene separately and for the two diagnosis atopic eczema and respiratory allergy.

### 3.7. Haplotype Analysis

Haplotypes of rs102275-rs174448 were constructed and are shown in Table 8. The LD between the two *FADS* SNPs was (*r*^2^ = 0.53, *D’* = 0.75) and LD between the two *ELOVL* SNPs was (*r*^2^ = 0.43, *D’* = 1.0). While haplotype regression was significant for the same fatty acids as rs102275, when the SNPs were included in the same model only rs102275 stood out as significant.

**Table 8:** Haplotypes of rs102275-rs174448 and of rs2236212-rs17606561.

	Haplotype	Frequency
*FADS* gene cluster	rs102275-rs174448	
	C–G	0.292
	T–G	0.055
	C–A	0.072
	T–A	0.581
*ELOVL2* gene	rs2236212-rs17606561	
	A–C	0.238
	G–C	0.185
	G–G	0.577

## 4. Discussion

Both desaturase and elongase enzymes are involved in the endogenous production of long chain PUFAs (≥ 20 carbon atoms) of the *n*-6 and *n*-3 series from precursors of 18 carbon atom chain-length. To our knowledge, this is the first study to compare the effect of polymorphisms in genes encoding for both these enzymes, the *FADS* genes and the *ELOVL2* gene, on fatty acid profiles at two time points, at birth and at 13 years of age in the same children. This is also the first study to analyze the association between allergy development and polymorphism in the *ELOVL2* gene. The results showed that polymorphism in the *FADS* genes and, nominally, in the *ELOVL2* gene were associated with the proportions of some *n*-6 PUFAs in serum phospholipids. Thus, minor allele carriers were found to have lower proportion of the long-chain product in the serum phospholipids and increased proportions of the substrate. The desaturases and elongases works on both *n*-3 and *n*-6 PUFAs, but the SNPs investigated here were mainly found to affect the *n*-6 PUFA levels, as previously shown [15,22].

The potential differential effect of polymorphism on PUFA proportions at the two time points are not assessed. Other factors than polymorphism in the *FADS* and *ELOVL* genes influence the proportions of PUFA differently at birth and at adolescent state, also difference in storage time between cord serum samples and adolescent serum samples might affect the results of the PUFA measurements. The PUFA levels in cord blood might be partly affected by the efficiency of the transport of PUFAs across the placenta from the maternal to the fetal circulation. While the PUFA levels in adolescence might be more affected by e.g., differences in dietary intake. We have previously shown that fish intake correlated to proportions of *n*-3 long chain PUFAs in these subjects at 13 years of age [36]. In contrast to our findings, a previous study [22] found stronger correlations between *FADS* polymorphisms and long chain PUFA proportions in serum at seven years of age than at birth.

Previous studies have revealed that single nucleotide polymorphism (SNPs) in the *FADS* gene cluster affect the proportions of PUFA and long chain PUFA in human tissue [7,8,9,10,11,12,13,14,15,16,17,18,19,20,21,22,23]. Polymorphism in the *FADS* genes carried by the minority of subjects have generally been associated with enhanced blood proportions of the *n*-6 and *n*-3 substrate fatty acids and decreased proportions of the product fatty acids, similar to the results in this study. However, the associations between *FADS* alleles and cord blood proportions of PUFAs has only been investigated in a single birth-cohort, the ALPSAC cohort from which two studies derive [15,22]. Steer *et al.* [22] investigated genetic variation in both the mother and the child in two *FADS2* SNPs, rs1535 and rs174575, in relation to fatty acids at three time points, *i.e.*, in maternal red blood cells during pregnancy, in cord plasma at birth and in child plasma obtained at seven years of age. Lattka *et al.* [15] analyzed associations between 17 additional SNPs in the whole *FADS* gene cluster and cord plasma fatty acid proportions in the same cohort, one of which, rs174448, was analyzed in our study. In both studies investigating the ALPSAC cohort, the minor alleles were associated with reduced amounts of the products and enhanced amounts of the precursors, and stronger associations between *FADS* SNPs and the *n*-6 series of PUFAs, than with the *n*-3 series of PUFAs [15,22]. Further, similar to our findings, rs174448 was associated with lower proportions of 20:3 *n*-6 and higher proportions of 20:4 *n*-6 and 22:4 *n*-6 in cord serum phospholipids, but no effect on *n*-3 PUFAs [15].

The pathway of endogenous production of long chain *n*-6 and *n*-3 PUFAs involves another gene family, elongases, which elongates the fatty acid chain. In the present study two *ELOVL2* SNPs were analyzed: rs17606561 and rs2236212. Minor allele carriers of rs17606561 had decreased proportions of arachidonic acid (20:4 *n*-6) and minor allele carriers of rs2236212 had a lower ratio of DHA over DPA (22:6 *n*-3/22:5 *n*-3) in cord serum. However, none of these associations persisted correction for multiple testing. Similar results, with increased proportions of DPA and decreased proportions of DHA in minor allele carriers of rs2236212 were found by Lematire *et al.* [25] in adults. To our knowledge no previous studies have evaluated the association between *ELOVL2* polymorphism and fatty acid proportions in cord serum phospholipids.

The SNPs in the present study were chosen to represent all SNPs in the *FADS* gene cluster or the *ELOVL* gene family that have shown significant association to serum phospholipids in two genome-wide association studies [24,25]. Lematire *et al.* found that minor allele carriers of rs102275 had increased proportions of α-linolenic acid (18:3 *n*-3) and decreased docosapentaenoic acid (22:5 *n*-3, DPA) proportions. Minor allele carriers of rs174448 had increased proportions of α-linolenic acid, decreased eicosapentaenoic acid (20:5 *n*-3, EPA) proportions and decreased docosahexaenoic acid (22:6 *n*-3, DHA) proportions [25]. We did not replicate the results found by Lematire *et al.* regarding *n*-3 PUFAs since we only found significant associations between *n*-6 PUFAs and these SNPs. Demirkan *et al.* [24] analyzed the genome-wide association to circulating phospholipid concentrations and found both amounts and proportions of many phospholipids to be associated to *FADS1* and *ELOVL2* polymorphism on a genome wide level. Demirkan *et al.* [24] did not report the fatty acid composition in the phospholipids and our results are therefore not comparable. A recent Genome Wide Association Study showed rs102275 and rs174448 to have a genome wide significant association to a range of long chain fatty acids, e.g., 20:3 *n*-6 and 20:4 *n*-6 [23]. This study also found SNPs in the *ELOVL2* gene to be associated with 22:5 *n*-3 (DPA) [23], however none of the *ELOVL* SNPs were analyzed in our study.

Two SNPs in the *FADS* gene cluster were analyzed here: rs102275 and rs174448. The SNP rs102275 is situated intergenic downstream of *FADS1* and is in strong LD with other SNPs in *FADS1* and *FADS2* genes*.* The strongest association of the rs102275 polymorphism was found for the product/precursor ratio for the Δ-5-desaturase in the *n*-6 pathway. The other analyzed SNP in the *FADS* gene cluster, rs174448, is situated intergenic between *FADS2* and *FADS3*. When the two *FADS* SNPs were added together in the linear regression models with fatty acids, only rs102275 was significant. The same was true when the haplotypes of rs102275-rs174448 were added. This indicates that the association to rs174448 is just a consequence of the LD with rs102275. These findings are also in accordance with the findings by Ameur *et al.* [44] who found two haplotype blocks in the *FADS* region, where rs102275 is situated in their LD block 1 that had the strongest association to long chain PUFAs and rs174448 is in their LD block 2 with weaker association. Additionally, the C and T alleles of rs102275 corresponds to the haplotypes they denoted A and D respectively in block 1 and our association pattern with the *n*-6 PUFAs is in agreement with theirs.

The subjects in this study were selected based on their allergic manifestation at 13 years of age. The power of this study may be limited by the relative small population size, however, the prospective birth cohort design allowed us to select very clear cases with only one allergic manifestations as well as non-allergic controls that had not been sensitized nor had any allergic symptoms in any of the follow-ups at 1, 4, 7, or 13 years of age. We aimed for two group of subjects that had allergic symptoms from one organ only, *i.e.*, atopic eczema or respiratory allergy, since the literature suggests that the association between PUFAs and allergy may differ in different allergic manifestations [36,45,46,47]. Here, an association between SNPs in the *FADS* gene cluster and allergy were found only for subjects with atopic eczema and not for subjects with respiratory allergy. The minor alleles of rs102275 (C) and the minor allele of rs174448 (G) were nominally protective against developing atopic eczema. However, as expected, the significance of rs102275 and rs174448 with atopic eczema disappeared when any of the five fatty acids that are associated with eczema as well as the SNP are added to the logistic model, suggesting that the association between the minor *FADS* allele and protection from atopic eczema development acts via a reduced capacity to elongate precursor *n*-6 PUFAs to arachidonic acid (20:4 *n*-6).

The association between SNPs in the *FADS* gene cluster and allergic diseases has been reported in three German (ECRHS, LISA, GINI) and one Dutch (KOALA) study with inconsistent results [13,17,48,49]. Schaeffer *et al.* found that adult minor allele carriers of several SNPs had lower prevalence of self-reported allergic rhinitis (*n* = 76) and atopic eczema (*n* = 49) in the German ECRHS study [13]. To the contrary, Rzehak *et al.* that reported results from the two birth cohort studies, KOALA and LISA, found that minor allele carriers of several SNPs in the *FADS* gene cluster had a higher prevalence of parental reported eczema at two years of age (*n* = 166) [17]. However, the association between polymorphism in the *FADS* genes and allergy has also been reported for the LISA study when the participants were 6 [49] and 10 years old [48], together with subjects from the GINI study. At these two later ages there were no longer any association between polymorphism and asthma, bronchitis, eczema or hay fever at 6 years of age [49] or at 10 years of age [48]. Further studies are needed to confirm the protective effects of the minor allele in *FADS* genes polymorphism found on atopic eczema in this study and on atopic eczema and allergic rhinitis in the study by Schaeffer *et al.* [13].

We have previously published the association between serum proportions of fatty acids and allergy in the same subjects [33,36], this paper additionally adds the polymorphisms. We reported that high proportions of long chain PUFAs at birth, in cord serum, were associated with allergy development [33]. At 13 years of age, when the allergic disease was manifest, there was no longer any correlation between atopy and serum PUFA proportions [36], which suggests that the PUFA milieu is important chiefly during early infancy when the naïve immune system of the infant is primed as foreign antigens are encountered. The mechanism by which high proportions of long-chain PUFAs in cord blood increases the risk of allergy development is unknown. Arachidonic acid is a precursor for prostaglandin E_2_ (PGE_2_) that promotes maturation of dendritic cells into a phenotype that supports Th2 development [50]. Furthermore, PUFAs counteract T cell activation and production of interferon-gamma; if the immune system is not activated in early infancy because of low microbial stimulation, or a mileu rich in PUFA, immune maturation might be hampered. Interestingly, the other allergic phenotype, respiratory allergy, which was also associated with a higher proportion of long chain PUFAs in cord serum [33] were not associated with having a different genotype. However, we found the strongest risk of respiratory allergy development to be connected with *n*-3 PUFAs [33] and these were obviously not strongly affected by the gene polymorphisms studied here. This might suggest that *n*-3 PUFAs in the fetus are derived chiefly from placental transport from the maternal circulation and are less dependent on synthesis by the fetus, and hence not as strongly associated to genetic variation in the *FADS* and *ELOVL* genes.

## 5. Conclusions

In the present study we found polymorphisms in the *FADS* gene cluster to be associated with lower proportions in cord serum phospholipids of arachidonic acid and adrenic acid (the products of the reaction catalyzed by the desaturase enzyme) and higher proportions of dihomo-γ-linolenic acid (the precursor). The results were similar, but less pronounced in the subjects when investigated at 13 years of age. We also found that polymorphisms in the *FADS* gene cluster were nominally associated with reduced risk of developing atopic eczema. The association between a reduced capacity to desaturase *n*-6 PUFAs due to *FADS* polymorphisms and reduced risk for eczema development, could indicate a pathogenic role for long-chain PUFAs in allergy development. Polymorphisms in the *ELOVL2* gene were nominally associated with decreased proportions of arachidonic acid in cord serum and decreased ratio of product over substrate in cord and adolescent serum, but not to the risk of developing allergic disease.
